# Synthetic Organic Electrochemistry in Ionic Liquids: The Viscosity Question

**DOI:** 10.3390/molecules16075963

**Published:** 2011-07-18

**Authors:** Steven Bornemann, Scott T. Handy

**Affiliations:** 1 Department of Chemistry, Binghamton University, Binghamton, NY 13902, USA; 2 Department of Chemistry, Middle Tennessee State University, Murfreesboro, TN 37132, USA

**Keywords:** room temperature ionic liquid, carbamate oxidation, constant current, viscosity, organic electrosynthesis

## Abstract

Ionic liquids are obvious candidates for use in electrochemical applications due to their ionic character. Nevertheless, relatively little has been done to explore their application in electrosynthesis. We have studied the Shono oxidation of arylamines and carbamates using ionic liquids as recyclable solvents and have noted that the viscosity of the medium is a major problem, although with the addition of sufficient co-solvent, good results and excellent recovery and recycling of the ionic liquid can be achieved.

## 1. Introduction

One of the universal truths regarding electrochemistry is that the reaction medium must be capable of carrying a charge. In general this has been accomplished by employing a supporting electrolyte (a salt) that is soluble in the reaction medium. While highly successful, this does limit the “greenness” of electrochemistry by necessitating a separation event and creating a waste material. Further, the supporting electrolyte is often one of the most expensive materials used in synthetic electrosynthesis. As a result, it would be of great potential benefit if the supporting electrolyte could be both readily separable from the reaction products and also readily recycled.

**Figure 1:**
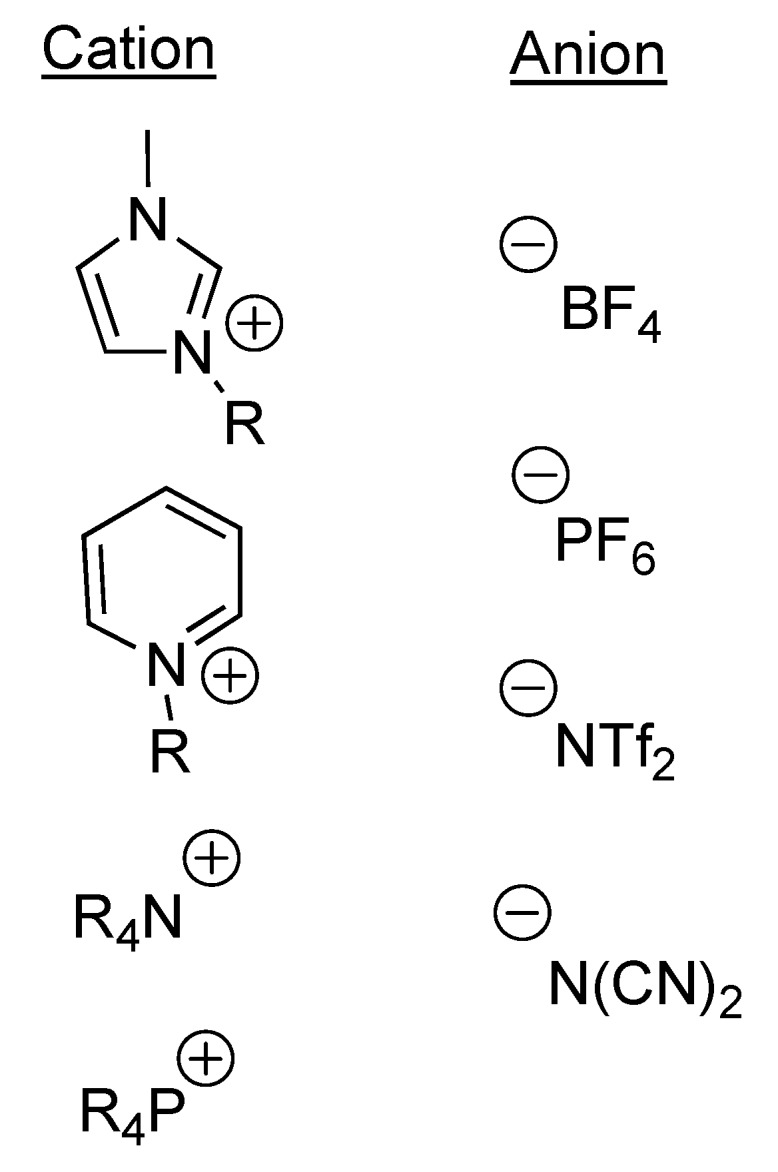
Representative ionic liquid components.

In recent years, the advent of hydrolytically stable room temperature ionic liquids (RTILs) has provided new and unusual solvents for reactions [1,2,3]. RTILs can be simply characterized as salts that are liquid at or below room temperature. Thus they are comprised of a cation (usually a relatively large organic cation) and an anion (usually a weakly coordinating inorganic anion), several examples of which can be seen in Figure 1. They have attracted increasing attention as potential green solvents over the last several years since they are, for all practical purposes nonvolatile and thus readily recyclable [4]. Additionally, they appear to be favorable media for a wide range of organometallic reactions and are often capable of providing extra stabilization to catalysts, thereby rendering them recyclable as well [5,6]. We have been interested in the use of RTILs in organic synthesis as well as the preparation of new RTILs [7,8,9,10,11,12]. During the course of these efforts, we have also been struck by the fact that, as salts, RTILs should provide a good, conducting medium in which to perform electrosynthesis. We have not been alone in this observation, as there have been a few other reports of the use of RTILs in organic electrosynthesis [13,14]. Surprisingly, though, this activity has been largely limited to a few select areas of organic electrosynthesis: polymerizations [15,16,17], fluorinations [18,19,20,21,22,23], oxidations [24,25,26,27,28], reductions [29,30], transition-metal catalyzed couplings/dehalogenations [31,32,33,34,35], and electrogenerated bases [36,37,38,39,40]. In this paper, we wish to outline our results on Shono-type carbamate oxidations as well as some observations regarding the impact that solvent viscosity has on the outcome of these (and possibly other) reactions.

## 2. Results and Discussion

**Scheme 1:**
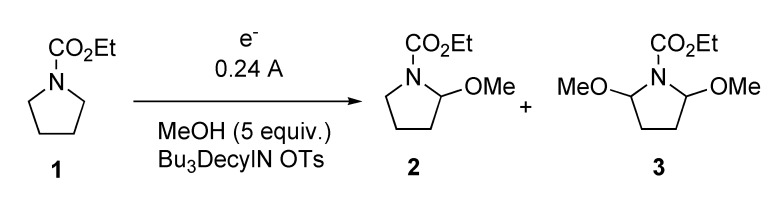
Shono oxidation in tributyldecylammonium tosylate.

The Shono-type oxidation of carbamates is certainly one of the simpler and yet more versatile electrochemical transformations [41]. It has been performed on a wide range of carbamate, amide, and even aniline substrates to afford either alkoxylated or alkene products. Both of these products can in turn be further transformed into a wide range of new products and have found a number of applications in natural and non-natural products synthesis. The simplicity of the reaction stems from the fact that it is performed under constant current conditions, meaning that the apparatus and set-up for the reaction is quite simple. Armed with a basic power supply, initial attempts were made to transform carbamate **1** into methoxylated product **2** using tributyldecylammonium tosylate as the solvent along with 5 equivalents (5 volume %) of methanol (Scheme 1). Under these conditions, the reaction mixture rapidly darkened and carbamate **1** was not consumed until over 3.5 F/mol of current was passed. Ultimately, traces of the desired product **2** could be isolated as well as some dimethoxylated product **3**. The RTIL itself was a black, syrupy mixture that contained numerous impurities based on ^1^H NMR analysis of the recovered RTIL.

**Figure 2:**
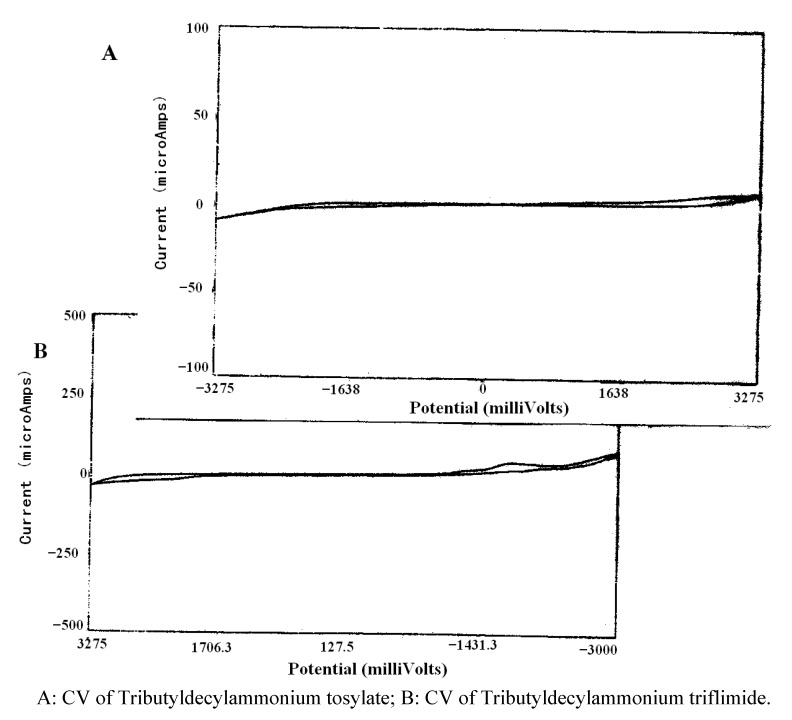
Electrochemical windows of the tributyldecylammonium RTILs.

The darkening of the RTIL was unexpected given its wide electrochemical window (as determined by CV). Indeed, the CV’s of all three RTILs employed in this study displayed little reactivity between −2.8 and 3.2 V. Representative CV’s of tributyldecylammonium tosylate and the corresponding triflimide can be found in Figure 2.

This initial result was not terribly promising, since the RTIL could not be recycled. Interestingly, though, by increasing the volume% of methanol, better results could be obtained (Table 1). Although a volume % of 33 still resulted in darkened RTIL that could not be recycled, increasing this further to an equivolume mixture of ionic liquid and methanol finally resulted in a good yield of oxidation product **2** after passing 2.5 F/mol of current. Further, the ionic liquid was pale yellow and readily recovered at the end of the reaction. Further increasing the percentage of methanol resulted in minor improvements in terms of yield and the physical state of the recovered ionic liquid.

**Table 1:** Effect of increasing volume% of methanol on the oxidation of carbamate **1** in tributyldecylammonium tosylate.

Entry	Volume% MeOH	% Yield of 2	State of RTIL	Viscosity (cP)
1	33	0	Dark black	120
2	50	88	Pale yellow	15
3	67	90	Clear	7

At this point, it was clear that the use of tributyldecylammonium tosylate as the ionic liquid solvent was not going to prove satisfactory. As will be discussed further, it was suspected that the viscosity of the medium might be playing a role in the observed results, so we opted for a less viscous ionic liquid —Tributyldecylammonium triflimide. Using this much less viscous ionic liquid, low volume%’s of methanol still resulted in significant darkening (decomposition) of the ionic liquid and low yields of the desired oxidation product **2** (Table 2). However, by the time the volume % reached 33, a pale yellow reaction mixture resulted from which 89% of **2** could be isolated and the RTIL recycled in further reactions (Table 2, entry 3). Thus, after passing 2.2 F/mol of current, the methanol was removed via evaporation and the product and any remaining starting material were then removed by extraction with diethyl ether. After chromatography, product **2** was isolated in 89% yield, along with 8% of the starting carbamate **1**. The resulting RTIL layer was pale yellow and could be reused in further reactions after drying in vacuo at 5 °C overnight (Table 2, entries 5–7). Increasing the volume % of methanol further did not result in any additional benefit (Table 2, entry 4).

**Table 2:** Oxidation of carbamate **1** in tributyldecylammonium triflimide.

Entry	Volume% MeOH	% Yield of 2	State of RTIL	Viscosity (cP)
1	10	10	Black	150
2	20	45	Dark brown	50
3	33	89	Pale yellow	10
4	50	93	Clear	5
5 ^a^	33	89	Pale yellow	
6 ^b^	33	91	Pale yellow	
7 ^c^	33	93	Pale yellow	

^a^ RTIL recycled from entry 3; ^b^ RTIL recycled from entry 5; ^c^ RTIL recycled from entry 6.

Although successful, we were still unsatisfied with the amount of methanol that was required for these reactions. In an effort to further minimize the amount of methanol employed, we sought an even less viscous RTIL that was still readily available and electrochemically stable. To this end, 1-ethyl-3-methylimidazolium triflimide was selected. (Table 3) Gratifyingly, the volume % of methanol required for clean reaction did decrease to 25%. Under these conditions, good conversion to product **2** was observed and the RTIL could be readily recycled after drying in vacuo overnight.

Using these same conditions, a number of carbamates could be oxidized in similar fashion and in good yield (Table 4). Isolation of the products and recycling of the RTIL was straightforward. Curiously, anilines (such as dimethyaniline) failed completely under these conditions, instead affording only dark mixtures of numerous products from which the anticipated oxidation product could not be separated.

**Table 3:** Oxidation of carbamate **1** in 1-ethyl-3-methylimidazolium triflimide.

Entry	Volume% MeOH	% Yield of 2	State of RTIL	Viscosity (cP)
1	5	0	Black	25
2	10	4	Dark orange	16
3	20	9	Dark yellow	10
4	25	83	Clear	6
5 ^a^	33	89	Pale yellow	
6 ^b^	33	91	Pale yellow	
7 ^c^	33	93	Pale yellow	

^a^ RTIL recycled from entry 4; ^b^ RTIL recycled from entry 5; ^c^ RTIL recycled from entry 6.

**Table 4:** Oxidation of various carbamates in 1-ethyl-3-methylimidazolium triflimide.

Entry	Carbamate	% Yield of Methoxylated Product
1	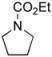	95
2	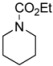	89
3	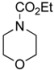	81
4	N,N-diethyl	No reaction

## 3. Experimental

All reagents and solvents were used as received. An Epsilon system from BAS was employed for all CV experiments. A standard power supply used for gel electrophoresis from VWR was employed for all Shono oxidation experiments. A Bruker NMR spectrometer (360 MHz at ^1^H) was used to collect all NMR spectra.

### 3.1. Decyltributylammonium Tosylate

#### 3.1.1. Decyl Tosylate

In a round-bottom flask, 33.8 g (0.214 mol) of decyl alcohol was added to pyridine (100 mL) and allowed to stir for a few minutes. Tosyl chloride (48.85 g, 0.257 mol) was slowly added while the flask was cooled in an ice water bath. The solution was allowed to stir under nitrogen for 3 h. The product was diluted with hexanes (800 mL) and passed through a small plug of silica (30% ethyl acetate in hexane). The resulting solution was then washed three times with HCl (1N, 200 mL) and once with distilled water (200 mL). The organic layer was dried with MgSO_4_, and the remaining solvent was removed under reduced pressure to afford 52.3 g of decyl tosylate (yield 84%). ^1^H-NMR (360 MHz, CDCl_3_): δ 7.77 (d, 2H, *J* = 10.8), 7.32 (d, 2H, *J* = 7.2 Hz), 3.98 (t, 2H, *J* = 3.9 Hz), 2.43 (s, 3H), 1.64 (m, 2H), 1.23 (m, 14H), 0.086 (t, 3H, *J* = 7.92 Hz).

#### 3.1.2. Decyltributylammonium tosylate

Decyl tosylate (47.44 g, 0.152 mol) was dissolved in ethanol (50 mL) in a round-bottom flask. To this was added of tributylamine (38.11 mL, 0.16 mol) with stirring. The solution slowly brought to 75–80 °C and heated overnight. The ethanol was removed under reduced pressure, and the salt was washed three times with hexanes. The residual hexanes was removed at reduced pressure, and the product was dried overnight under vacuum at 70 °C to produce 72 g (88%) of DTAT as a pale yellow very viscous liquid, which solidifies over the period of several days. ^1^H-NMR (360 MHz, CDCl_3_): δ 7.60 (d, 2H, *J* = 9.72 Hz), 6.98 (d, 2H, *J* = 10.8 Hz), 3.00 (m, 6H), 2.83 (m, 2H), 2.19 (s, 3H), 1.48 (m, 6H), 1.36 (m, 2H), 1.15 (m, 18H), 0.75 (m, 14H). ^13^C-NMR: (90 MHz, CDCl_3_) δ 142.0, 140.6, 130.1, 129.3, 60.3, 60.1, 31.9, 29.6, 29.5, 29.4, 29.1, 29.0, 24.5, 24.3, 22.7, 21.3, 21.2, 14.1, 13.9.

#### 3.1.3. Decyltributylammonium bis(trifluoromethanesulfonyl)imide

In a round-bottom flask, tributylamine (37 g, 0.20 mol) was stirred with acetonitrile (47 mL). Bromodecane (44.2 g, 0.20 mol) was then added dropwise to this solution. The mixture was stirred at 70 °C for 48 h after which the solvent was removed under reduced pressure. The product was washed three times with hexanes and then dried over night under reduced pressure to afford 69.02 g (85%) decyltributylammonium bromide, as an orange viscous liquid. Decyltributylammonium bromide (47.81 g, 0.118 mol) was dissolved in distilled water (154 mL). Separately lithium bis(trifluoro-methanesulfonyl)imide (34.2 g, 0.120 mol) was dissolved in distilled water (25 g). The two aqueous solutions were mixed together and then stirred at room temperature for at least three hours. The product (organic phase) was separated from the aqueous phase and was washed with distilled water three times to remove any water-soluble impurities. The product was then stirred with activated charcoal and filtered with neutral alumina. The final product was dried overnight under vacuum at 70 °C to afford 64 g (92%) of the product as a pale orange viscous liquid. The product was determined to be halide-free via the method described by Seddon [42]. ^1^H-NMR (360 MHz, CDCl_3_): δ 3.15 (m, 8H), 1.59 (m, 8H), 1.31 (m, 18H), 0.935 (m, 14H). ^13^C-NMR (90 MHz, CDCl_3_) δ 137.2 (q, *J* = 278 Hz), 60.4, 60.0, 31.8, 29.8, 29.6, 29.5, 29.1, 29.0, 24.4, 22.6, 21.3, 21.1, 14.0, 13.9.

#### 3.1.4. 1-Ethyl-3-Methylimidazolium bis(trifluoromethanesulfonyl)imide [43]

To a round-bottom flask were added 1-methylimidazole (38.84 mL, 0.488 mol) and ethylbromide (36.00 mL, 0.488 mol). The solution was stirred at 75–85 °C for 72 h. The product was washed three times with ethyl acetate. The residual ethyl acetate was removed at reduced pressure to afford 1-ethyl-3-methylimidazolium bromide as a very hard, white solid. 1-Ethyl-3-methylimidazolium bromide (22.5 g, 0.118 mol) was dissolved in distilled water (154 mL). Separately lithium bis-(trifluoromethanesulfonyl)imide (34.2 g, 0.120 mol) was dissolved in distilled water (25 g). The two aqueous solutions were mixed together and then stirred at room temperature for at least 3 h. The product (the organic phase) was separated from the aqueous phase and was washed with distilled water three times to removed any water-soluble impurities. The product was then stirred with activated charcoal and filtered through neutral alumina. The final product was dried overnight under vacuum at 70 °C to afford and 41 g (88%) of the product as a colorless slightly viscous liquid. The product was determined to be halide-free via the method described by Seddon [42]. ^1^H-NMR (360 MHz, CDCl_3_): δ 8.82 (s, 1H), 7.27 (d, 2H, *J* = 7.2 Hz), 4.25 (q, 2H, *J* = 7.2 Hz), 3.95 (s, 3H), 1.58 (t, 3H, *J *= 7.2 Hz).

### 3.2. General Procedure for Preparing Carbamates

#### N-Carboethoxypyrrolidine

In a dry round-bottom flask CH_2_Cl_2_ (100 mL) was added to an equal volume of saturated aqueous sodium bicarbonate. Pyrrolidine (7.1 g, 0.100 mol) was added to the flask and allowed to stir in an ice bath. To this mixture, ethyl chloroformate (11.4 g, 0.105 mol) was added slowly while watching for heat evolution. The reaction was allowed to stir for 4 h after which the product (organic phase) was separated from the aqueous phase and was washed with distilled water three times to remove any water-soluble impurities. The organic layer was then dried with magnesium sulfate, filtered, and the solvent was removed *in vacuo* to afford 13.7 g (97%) of the carbamate as a colorless liquid with spectral properties consistent with those reported in the literature [41]. ^1^H-NMR (360 MHz, CDCl_3_): δ 4.02 (q, 2H, *J* = 7.2 Hz), 3.24 (m, 4H), 1.74 (m, 4H), 1.14 (t, 3H, *J* = 7.2 Hz). All carbamates were prepared according to this general method using the corresponding amines.

*N-Carboethoxypiperidine* [44]. ^1^H-NMR (360 MHz, CDCl_3_): δ 4.03 (q, 2H, *J* = 7.2 Hz), 3.32 (t, 4H, *J* = 7.2 Hz), 1.46 (m, 6H), 1.17 (t, 3H, *J* = 7.2 Hz).

*N-Carboethoxymorpholine* [41]. ^1^H-NMR: (360 MHz, CDCl_3_) δ 4.01 (q, 2H, *J* = 7.2 Hz), 3.514 (t, 4H, *J* = 5.04 Hz), 3.33 (t, 4H, *J* = 5.04 Hz), 1.132 (t, 3H, *J* = 7.2 Hz).

### 3.3. Electrochemical Oxidation of N-Carboethoxypyrrolidine

#### The Control [45]

Into a 20 mL undivided cell fitted with a glassy carbon anode (the anode dimensions were 2.4 cm × 2.4 cm though only 1 cm^2^ was submerged in the reaction solution) and a platinum wire cathode (2 mm diameter) were placed *N*-carboethoxypyrrolidine (2.00 g, 0.014 mol) and tetraethylammonium *p*-toluenesulfonate (Et_4_NOTs, 0.11739 g, 0.00039 mol) as electrolyte and methanol (12 mL) as the solvent. While being stirred with argon bubbling, a constant current of 0.240 A was passed through the cell, which was externally cooled with water. After 2.34 F/mol of electricity was passed (214 min), the solvent was removed *in vacuo*, and the product was separated from the electrolyte via distillation to afford the product in 82% with spectral properties consistent with those reported in the literature. ^1^H-NMR (360 MHz, CDCl_3_): δ 5.07 (m, 1H), 3.93 (q, 2H, *J* = 7.2 Hz), 3.26 (s, 3H), 3.17 (m, 2H), 1.78 (m, 4H), 1.18 (t, 3H, *J* = 7.2 Hz).

### 3.4. Using Ionic Liquid as Solvent/Electrolyte

Into a 20 mL undivided cell fitted with a glassy carbon anode (the anode dimensions where 2.4 cm × 2.4 cm though only 1 cm^2^ was submerged in the reaction solution) and a platinum plate cathode (the cathode dimensions where 2.48 cm × 2.45 cm though only 1 cm^2^ was submerged in the reaction solution) were placed *N*-carboethoxypyrrolidine (0.70 g, 0.005 mol) and a solution of EMIM NTf_2_ (2.6 mL) with methanol (0.8 mL) as the solvent/electrolyte. While being stirred with argon bubbling, a constant current of 5 mA was passed through the cell, which was externally cooled with water. After 800 min, the methanol was removed under reduced pressure and the product was separated from EMIM NTf_2_ via distillation to afford the product in 89% yield along with recovered starting material. The current efficiency was 2.35 F/mol. The salt was then prepared for recycling by heating, at reduced pressure, to 100 °C for 2 h to remove any residual solvent or water absorbed from the air.

*1-Methoxy-N-carboethoxypiperidine*. This same procedure was repeated with recycled salt using *N*-carboethoxy piperidine to afford the methoxylated product in 95% yield with a 2.62 F/mol efficiency with spectra consistent with those reported in the literature [46]. ^1^H-NMR (360 MHz, CDCl_3_): δ 5.30 (m, 1H), 4.16 (q, 2H, *J* = 7.2 Hz), 3.37 (s, 3H), 3.26 (m, 2H), 1.98 (m, 2H), 1.54 (m, 4H), 1.31 (t, 3H, *J* = 7.2 Hz).

*1-Methoxy-N-carboethoxypiperidine*. This same procedure was repeated with recycled salt using *N*-carboethoxy morpholine to afford the methoxylated product in 81% yield with a 3.05 F/mol efficiency with spectra consistent with those reported in the literature [47]. ^1^H-NMR (360 MHz, CDCl_3_): δ 5.10 (m, 1H), 4.18 (q, 2H, *J* = 7.2 Hz), 3.85 (s, 3H), 3.80 (m, 2H), 3.50 (m, 2H), 3.3 (m, 2H), 1.25 (t, 3H, *J* = 7.2 Hz).

### 3.5. Viscosity Measurements

All viscosities were determined using a Brookfield DVE viscometer. Reported values are the average of three measurements. Note that no temperature control module was available, so the temperature of the samples did vary slightly from between 20 and 22 °C.

## 4. Conclusions

During this course of this research, it became quite apparent that the high viscosity of ionic liquids is a definite problem for electrosynthesis. This feature has not been observed in most of the previous studies, because they were conducted under controlled potential conditions. Indeed, in one of the previous reports employing constant current conditions (as was done in the present study), Barhdadi and co-workers obtained poor results unless a co-solvent (DMF in their case) was employed to decrease the viscosity of the reaction medium [34]. What we suspect is happening is that the higher viscosity of the ionic liquid slows the rate of diffusion of the starting material to and from the electrode surface. Since the Shono oxidation occurs at or very near the electrode surface, the slowed diffusion results in a situation in which there is not enough starting material present to react with the current that is being generated. As a result, the next most easily oxidized species will react, thus leading first to over oxidation product **3** and then to oxidation of the ionic liquid.

This over oxidation problem can be limited by the use of VOC co-solvents, but that, obviously, reduces the value (in terms of being environmentally friendly) of using ionic liquids. The other option is to employ even less viscous ionic liquids. Although there is continuing improvement in this area, there is still a clear need for even less viscous ionic liquids (<10 cP based upon our observations) that still maintain a wide electrochemical window before their full potential in electrochemistry can be unleashed.
